# Gamma Activity Coupled to Alpha Phase as a Mechanism for Top-Down Controlled Gating

**DOI:** 10.1371/journal.pone.0128667

**Published:** 2015-06-03

**Authors:** Mathilde Bonnefond, Ole Jensen

**Affiliations:** Donders Institute for Brain, Cognition and Behaviour, Radboud University, Nijmegen, Netherlands; Federal University of Rio Grande do Norte, BRAZIL

## Abstract

Coupling between neural oscillations in different frequency bands has been proposed to coordinate neural processing. In particular, gamma power coupled to alpha phase is proposed to reflect gating of information in the visual system but the existence of such a mechanism remains untested. Here, we recorded ongoing brain activity using magnetoencephalography in subjects who performed a modified Sternberg working memory task in which distractors were presented in the retention interval. During the anticipatory pre-distractor period, we show that the phase of alpha oscillations was coupled with the power of high (80-120Hz) gamma band activity, i.e. gamma power consistently was lower at the trough than at the peak of the alpha cycle (9-12Hz). We further show that high alpha power was associated with weaker gamma power at the trough of the alpha cycle. This result is in line with alpha activity in sensory region implementing a mechanism of pulsed inhibition silencing neuronal firing every ~100 ms.

## Introduction

Neuronal oscillations are a prominent feature of brain dynamics and they can be detected in humans using various kinds of intra- and extracranial electrophysiological measures. Multitudes of brain rhythms have been observed in several species, ranging from frequencies below 1Hz to above 100Hz [1]. The functional role of oscillations in these frequency bands has been extensively studied. For instance, recent work suggests that alpha oscillations (7–14 Hz) are responsible for active functional inhibition of sensory regions in order to filter out incoming information [2–7]. In support of this hypothesis, it has been shown that alpha power is negatively correlated with the Blood Oxygen Level Dependent (BOLD) signal [8,9], the likelihood of experiencing a trancranial magnetic stimulation (TMS)-induced phosphene [10] and the firing rates in the somatosensory system of non-human primates [11]. Moreover, numerous studies in animals and humans have reported that detection of subthreshold stimuli, evoked BOLD signal, spiking and gamma activity (>30Hz) are all modulated by the phase of the alpha cycle [12–17]. Together, these results suggest a mechanism of ‘pulsed inhibition’ in which the transmission of sensory input is suppressed during parts of the alpha cycle [3,16]. Gamma oscillations (above 30Hz), on the other hand, are thought to reflect active processing of information [18,19]. In addition, several groups have reported interactions between alpha and gamma activity. More precisely, it has been shown that the power in various ranges of the gamma band (30-40Hz and above 60Hz) was coupled to the phase of alpha (4-7Hz) oscillations in sensory regions [12–14,20–22]. However, the coupling between alpha and ongoing gamma activity has mainly been observed during rest [13,21]. How such a coupling relates to specific cognitive states remains to be understood. Moreover, if alpha activity is associated with functional inhibition, more precisely with pulses of inhibition every ~100ms [3,4], then increase in alpha power should be associated with stronger pulses of inhibition. As a consequence, higher alpha power would be associated with a stronger suppression of gamma activity at a given alpha phase (e.g. the trough at which the pulse of inhibition might occur) while the activity at the opposite phase (e.g. the peak at which the inhibition level is back to baseline) should be relatively preserved.

In the present paper, we analyzed MEG data from a working memory task in which strong or weak distractors were introduced (in different blocks) during the retention interval. We analyzed cross frequency coupling between alpha and gamma frequency bands during the anticipation of a stimulus (here the distractor), a period in which most top-down controlled variation in the alpha band activity have been reported in the literature [7,23–26].

## Methods

### Ethics Statement

The study was conducted at the Donders Institute for Brain, Cognition and Behaviour, Radboud University Nijmegen the Netherlands with the general institutional ethics approval from the local ethics committee (Commissie Mensgebonden Onderzoek region Arnhem-Nijmegen, The Netherlands). All participants provided written informed consent in accordance with the declaration of Helsinki.

### Participants

We analyzed cross-frequency interactions from the data of seventeen healthy subjects (18–26 years of age, 16 females). The present results are a further analysis of previously published data [27]. However, the results presented in Bonnefond and Jensen [27]) were obtained from the analyses of the data of eighteen subjects, the data of one subject has been lost during a back-up process following the publication.

### Experimental Paradigm

**Fig 1:**
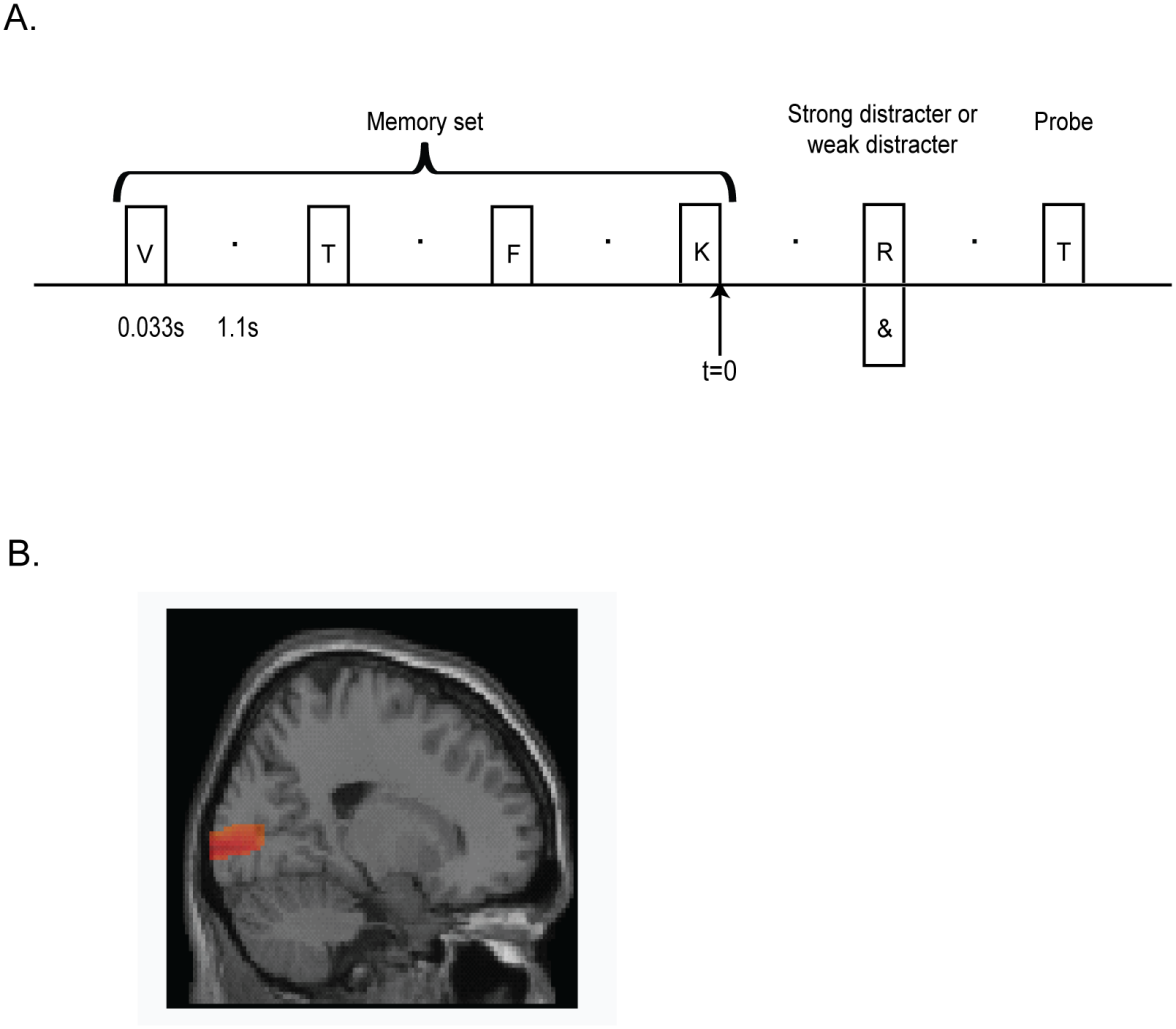
Design and region of interest. (A) Experimental design. In each trial, four consonants were presented sequentially for 33ms with an interstimulus interval of 1.1s. Subjects indicated whether the probe was similar to one of these letters or not (‘‘old” or ‘‘new” button press). In half of the blocks, a ‘strong’ (a letter) distractor was presented for 33m in the retention interval while, in the other half of the blocks, a ‘weak’ (a symbol, not represented here) distractor was presented. (B) Region of interest selected for the analysis [27]. MNI coordinates: -50, -70, 10.

We designed a Sternberg WM task including weak (a symbol, e.g. ‘{‘, ‘/’, ‘&’) or strong (a letter which was not part of the memory set) distractors in the retention interval. The experiment was divided into blocks where the same kind of distractor was used (4 blocks for each kind of distractor; 46 trials in each block). We used this approach so that participants could anticipate the type of distractor presented in each trial. The order of the blocks was randomized over subjects. For each trial, the four letters to be remembered were presented for 33ms (ISI = 1100 ms). After 1100ms, a distractor was presented for 33ms and after a 1100ms delay, the memory probe was displayed (Fig 1A). Participants were asked to ignore the distractor and indicate by button presses if the probe was ‘old’ or ‘new’. We focused the analysis on the time interval just prior to distractor items (0.6 to 1s). We chose this interval in order to avoid interference by activity induced by the memory or distractor items.

### Data Acquisition

Ongoing brain activity was recorded (sampled at 1200 Hz) using a whole-head MEG system with 275 axial gradiometers (VSM/CTF systems, Port Coquitlam, Canada). After the MEG acquisition, the structural MR images of the subjects’ brains were acquired using a 1.5 T Siemens Magnetom Sonata system (Erlangen, Germany). During the MR and MEG acquisitions, the same earplugs (now with a drop of Vitamin E in place of the coils) were used for coregistration of the MRI and MEG data.

### Data Analysis

The data was analyzed using the Matlab-based FieldTrip toolbox, developed at the Donders Institute for Brain, Cognition and Behaviour [28]. The data considered (trials) started 0.2s before the last memory items and until 0.2s after the probe onset. We only analyzed trials with a reaction time within interval mean +/- 2 standard deviations and without eye artifacts or jumps in the signal. In Bonnefond and Jensen (2012), we showed that both the power and the phase of alpha activity was adjusted prior to the strong distractor over occipital and left temporal cortices and further predicted the behavioral performance. In the present paper, we aim at analyzing the coupling between alpha and gamma activity in anticipation of the distractor items and during memory item and distractor processing. We did not conduct the analyses at sensor level as the contribution from different sources is mixed. Thus it reduces the chances of detecting more subtle cross-frequency interactions. We restricted the analyses to the signal observed in the left temporal cortex. More precisely, we selected the brain region, in the left temporal cortex, in which we previously observed strong alpha power difference between weak and strong distractor conditions (Fig 1B; MNI coordinates: -50, -70, 10;) [27]. Note however that similar results were found in the left and right occipital regions, i.e. the cross-frequency coupling reported here was not specific to the left temporal cortex. To extract the time course for each trial, from this region of interest, we used a linearly constrained minimum variance beamformer [29] on the detrended (non-filtered) data (entire trial interval, see above). A common filter was computed for both weak and strong distractor conditions. For each subject, a realistically shaped single-shell description of the brain was constructed, based on the individual anatomical MRIs. The lead field was calculated for the grid point of interest. Among the three dipoles obtained (each with a specific orientation), we selected the dipole with the maximum activity over all frequencies.

### Cross frequency coupling analysis

In order to quantify the coupling between gamma power and alpha phase in the pre-(strong and weak) distractor period, we used the Modulation Index (MI) [30].

We analyzed the coupling between the phase of the signal in the 7 and 20 Hz band (f_phase_; 1 Hz increment) and the amplitude of the signal between 25 and 140 Hz (f_ampl_; 2 Hz increment). We quantified the phase-amplitude coupling using the MI. We obtained the instantaneous phase estimate of the f_phase_ and amplitude estimate of the f_ampl_ using the angle and the magnitude of the Fourier-transform. Specifically, we performed time-frequency analyses using phase and power on the pre-distractor period (0.6 to 1s) using a fast Fourier transform (FFT) approach applied to the source data (the left temporal region of interest). This was done with a sliding time window (3 cycles long for f_phase_ and 4 cycles long for f_amp_; e.g. ΔT_phase = 3/f = 429 ms for 7Hz and 272 ms for 11 Hz; ΔT_amp = 4/f = 80ms for 50Hz). A Hanning taper was multiplied to the data prior to the Fourier transform. We obtained the f_phase_ and the f_amp_ for each time point in the time window of interest of each trial and then combined the values obtained. The large number of trials compensated for the relatively short time-window interval. The f_phase_ was then divided into 18-degree-bins (n_bins_ = 18). For each of these bins, the mean amplitude of the higher-frequency was computed and normalized by dividing it by the sum over all bins.

Finally, the modulation index (MI) was computed. This index assesses whether there is a measurable deviation of the amplitude distribution P from a uniform distribution U.

$$MI\;=\;\frac{D_{KL}(P,\;U)}{log(n_{bins})}$$

The Kullback–Leibler (KL) distance, D_KL_(P,U) = log(*nbins*)—H(*P*), where the Shannon entropy H of the distribution P, H(P) is equal to - $\sum_{ibin\;=\;1}^{N}P\left(\;i_{bin\;}\right)*log[P\left({\;i}_{bin}\;\right)]$. If P = U, i.e., the higher-frequency amplitude is the same for each bin of the lower-frequency phase, MI = 0. Conversely, a MI equal to 1 would be observed if P had a Dirac-like distribution (P = 1 for a given bin and 0 for all the other bins). In short, the magnitude of MI will reflect the degree of phase to power coupling.

### Alpha peak-aligned power spectra

To characterize the cross-frequency coupling, we aligned time-frequency representations to the peaks of alpha activity (at 11Hz which corresponds to the median frequency exhibiting a strong coupling with gamma activity as assessed by the MI analysis) detected in the 0.6 to 1s time window [31]:

1. We performed the time-frequency analyses of phase in the time window of interest (-0.6 to 1s) using a FFT approach applied to the region of interest. We used a sliding time window 3 cycles long (ΔT = 3/f; ΔT = 272 ms for 11 Hz) and a Hanning taper (ΔT long) was multiplied to the data prior to the Fourier transform.
2. We performed the time-frequency analyses of power (25 to 140 Hz; steps of 1 Hz) using the same parameters except that the duration of the sliding time window was 4 cycles long (e.g. ΔT = 4/f; ΔT = 80 ms for 50 Hz).
3. We detected the peaks (local maxima) of the 11Hz activity. To increase the likelihood of detecting peaks of actual oscillations, we only considered peaks that were surrounded by troughs (we independently detected the troughs of the 11Hz activity) at the time points expected regarding the frequency analyzed (e.g. at 11Hz the troughs are expected to be 45ms (+/-5ms) before and after the peak). We then aligned the time frequency representations on the detected peaks including one cycle of the modulating frequency duration before and after each peak (e.g. 160ms time windows for the 12Hz activity).
4. For each frequency, resulting power values were converted to relative power changes with respect to the mean value across the time window defined around the peak.
5. The alpha-peak-aligned power spectra were then averaged.

The modulation of the high-frequency power by a particular alpha phase (e.g., the peak) would be expressed as an increase or a decrease of the relative power values around the peak.

In some subjects, using the low gamma activity as a reference (30-40Hz), we observed that the alpha phase at which the gamma activity was significantly higher was different from the average observed over subjects; more precisely, gamma activity was higher at the alpha peak in the average and at the alpha trough in these subjects. As the excitability level associated with each observed phase of a signal is not meaningful per se as it depends e.g. on the orientation of the dipole localized, we multiplied the raw data by -1 for these subjects so they do not differ from the other subjects when we computed the alpha peak-aligned power spectra (4 subjects differed from the average).

### Statistical analyses of the cross-frequency coupling

To assess the reliability of the cross-frequency coupling, we created a surrogate distribution of MI for each combination of f_phase_ and f_amp_ by adding noise to the phase data in each trial. We repeated this procedure 500 times. For each subject, we thus obtained a distribution of MI which we then combined across subjects. Finally, the observed MI observed for each combination of f_phase_ and f_amp_ was transformed into a p value (t test) by comparing it to the mean and SD of the corresponding null distribution [32]. We only show the significant MI values on the figures. As we had specific hypotheses about alpha activity, we ran this analysis for f_phase_ between 8 and 14Hz. A false discovery rate (FDR) method was used to correct for multiple comparisons to avoid type I errors.

## Results

In each trial of this WM experiment, participants had to encode 4 items and determine whether a probe presented after the retention delay was among the letters encoded or not. Weak (symbols, e.g. ‘&’) or strong (another letter) distractors were always presented in the retention interval at the same time point (Fig 1A). The distractor types were grouped in blocks of trials such that the subject could anticipate the weak or strong distractors. The main behavioral effect we found was a longer reaction times for strong compared to weak distractor (725 +/- 35ms vs. 699 +/- 31 ms; p < 0.05) [27]. We focused the analysis on the pre-distractor period of the retention interval.

### High gamma power is coupled to alpha phase and anti-correlated with alpha power in the pre-distractor retention interval

We previously showed that alpha power is stronger in anticipation of strong compared to weak distractors [27,33]. Here we investigated whether alpha activity is associated with pulses of inhibition every ~100ms. We tested two predictions:

1. Gamma power should be coupled to alpha phase in the pre-distractor period, i.e. should be lower e.g. during trough than during peak of the alpha cycle.
2. The alpha power increase observed before the strong distractor should be associated to a decrease of gamma power at a specific phase, e.g. at the trough of alpha activity. More generally a change in alpha power regardless of the condition should be associated with such suppression.

**Fig 2:**
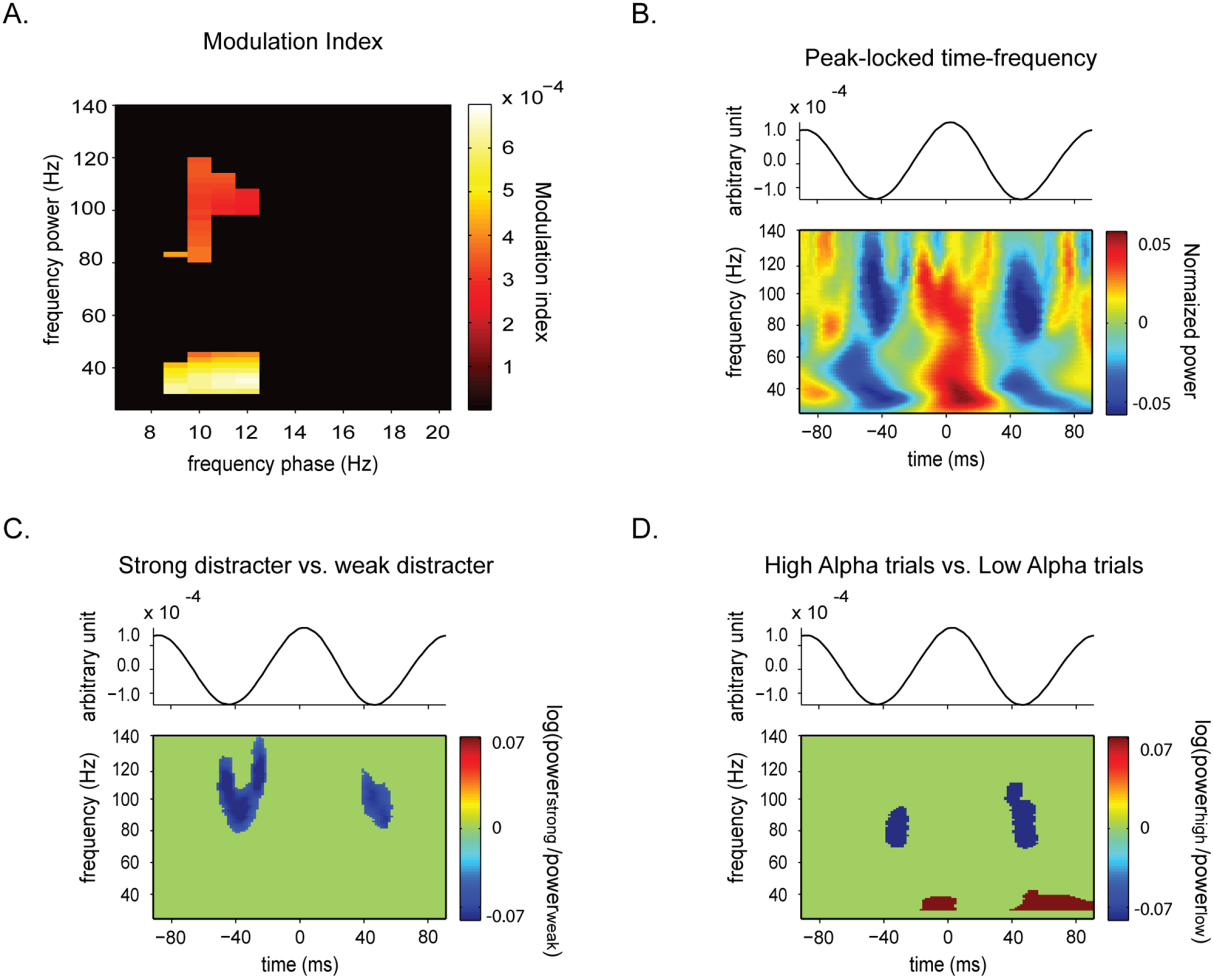
High gamma power-alpha phase coupling. (A) Modulation index (MI) analysis (pre-distractor period: 0.6–1 s). The analysis of the MI indicated a significant coupling (compared to the surrogate data) between the phase of alpha activity and the power of the low gamma activity (30-40Hz) and high gamma activity (80–120 Hz). (B) Normalized time-frequency representations of power for epochs of 180ms time-locked to the peaks of the 11Hz activity. The time-frequency representations were calculated for each epoch, averaged over the epochs and normalized relatively to the average power per frequency. This approach reveals the modulation of low and high gamma activity across the alpha cycle and supports the results obtained using the modulation index analysis. (C) Contrast of the peak-locked raw time-frequency representation between the strong and the weak distractor conditions. This contrast reveals a weaker high-gamma power (>80 Hz), specifically at the troughs of alpha activity, for the strong distractor condition. (D) Contrast of the peak-locked time-frequency representation for trials with high versus low alpha power in the strong distractor condition. This contrast reveals a similar result as the contrast between strong and weak distractor, i.e. a trough-specific decrease of the high-gamma power.

Regarding the first prediction, we indeed identified a significant cross-frequency coupling (as assessed by the modulation index) between alpha phase (9-12Hz) and low gamma (30–40 Hz) power and high gamma (80–120 Hz) power in the pre-distractor period (Fig 2A). The mean normalized TFRs (Fig 2B) aligned to the alpha peaks confirmed the coupling between alpha phase and gamma activity in the two frequencies. More specifically, the gamma power appeared smaller during alpha troughs than during the peaks (but see methods page 10 indicating that some subjects exhibited the reverse pattern).

Regarding the second prediction, we then compared the TFRs (not normalized) aligned to the alpha peaks for the strong versus weak distractor conditions. We performed statistical analyses using a cluster-based permutation test [34] which controls for multiple comparison issue. Significant clusters (i.e. set of significant adjacent points) in the time-frequency space (for frequencies from 30 to 120 Hz) were detected using a parametric test-statistic thresholded by an uncorrected p-value of 0.05. Then values in frequency-frequency tiles were reshuffled (500 times) randomly between the actual and the surrogate data and the maximum cluster size per permutation was stored to assess the distribution of maximal cluster sizes. Cluster size was defined as the sum of the t-values in that cluster. Cluster significance in a given contrast was assessed by comparing the cluster size with the distribution of the maximal cluster sizes across permutations. A cluster was considered to be significant at alpha = 0.05. This test controls for multiple comparisons since each cluster-level statistic is compared to the reference permutation distribution of the largest cluster-level statistic.

Our key finding was a decrease of gamma power, in the 80-120Hz band only at the trough of the alpha activity (Fig 2C).

In order to confirm that the differences observed for the contrast between strong and weak distractor was driven by the change in alpha power, we split the peak-locked TFRs obtained in the strong distractor condition into high and low alpha power trials and contrasted these two types of trials. We again observed a weaker high gamma power (>70Hz), specifically at the alpha troughs, in the high alpha power trials (Fig 2D). This result corroborates the result obtained for the contrast between strong and weak distractors. In addition, we detected a higher low gamma power (30-40Hz) at the peak of alpha activity which did not survive the cluster-based permutation test for the comparison between weak and strong distractors.

Despite the short stimulus presentation duration, we further observed sustained gamma activity (65-90Hz) during memory items and distractors processing ~350 ms after stimulus onset. We therefore conducted the cross-frequency coupling analyses for these intervals as well (S1 File). We found a striking inversion of the alpha phase at which gamma activity was expressed between the memory item and the distractor period (i.e. at the peak during the memory item processing and at the trough during the distractor processing; S1 Fig). These results should however be interpreted cautiously given the potential contamination by evoked fields (but see S1A Fig).

## Discussion

In the present paper, we examined the relationship between alpha activity and gamma activity in anticipation of a distractor in a Sternberg-like working memory task.

We found that gamma power in the 30-40Hz and in the 80-120Hz band were coupled to the phase of alpha activity, i.e. gamma power was lower at trough than at the peak of alpha activity (but see methods page 10). We further showed that higher alpha power was specifically associated with a lower high gamma (>80Hz) power in the alpha trough. This result is in line with the idea that alpha activity is associated with functional inhibition or more specifically with pulses of inhibition every ~100ms that disrupt ongoing activity [3,4,16,35,36]. This hypothesis predicts that the window of excitability should be smaller when alpha power is stronger possibly limiting the processing of incoming information. This decrease of the excitability associated with a stronger alpha power fits well with our previous results that showed that higher pre-distractor alpha power was associated with improved suppression of the distractor processing within and across participants [27]. An alpha phase-specific negative correlation between alpha and gamma power was also found in non-human primates during rest [14]. We here show that this mechanism generalizes to humans in a cognitive task.

The origin of the high gamma activity (above 80Hz) remains to be understood. Such a relatively high frequency has also been observed in the visual system during rest or during task in different studies [13,14,21] and has previously been proposed to reflect multi-unit activity [37,38]. We additionally found that the low gamma (30-40Hz) was higher for high alpha power compared to low alpha power, specifically at the peak of alpha activity. This low frequency could be associated with the generation of alpha activity, i.e. could drive it.

We further observed a different preferred alpha phase of the gamma power when comparing the memory encoding to the distractor presentation interval (see S1 Fig and S1 File). This finding suggests a mechanism of gating controlled by the gamma activity in relation to the phase of the alpha activity. The phase at which gamma activity occurs could allow the system to filter relevant from irrelevant information, beyond a difference in gamma power. This result relates to our proposition that alpha activity is a mechanism for prioritizing visual information, where more relevant stimuli are associated with a different phase than less relevant stimuli [35,39]. However, further investigations would be required to confirm this result and clearly dissociate the coupling between evoked vs. induced alpha activity in relation to the gamma power. Possibly this should be done in the context of longer stimulus intervals allowing to separate the induced activity from the evoked activity (also see discussion section in S1 File).

Finally, it would be of great interest to identify the brain regions and the mechanisms controlling posterior alpha and gamma activities (see e.g. [40,41–44]). Several papers have demonstrated that frontal regions might be involved in the control of alpha activity, in particular, the frontal eye field and the dorsolateral prefrontal cortex [27,45–48]. Saalman et al. [11] have further shown that the pulvinar might be involved in coordinating the top-down control of alpha activity initiated by frontal regions [49].

## Supporting Information

S1 Fig Stimulus induced gamma power-alpha phase coupling. (A) (top) Time-frequency analysis. This analysis indicated that the presentation of the memory item or of the distractor both induces an increase of gamma power in the 65-90Hz band and a decrease of alpha/beta power (8-30Hz) compared to a baseline period defined 200ms before the probe (middle) Time-frequency analysis of the evoked activity. This analysis revealed a strong evoked activity in a large frequency band. Only the activity in the theta and alpha band was sustained up to 300ms after the stimulus onset (bottom) Non-filtered evoked activity. The activity evoked by the memory item and distractor appeared similar. (B) Modulation index (MI) analysis (stimuli periods; memory item: 0.1–0.45s, distractor: 1.2–1.55s). The analysis of the MI indicated a significant coupling (compared to the surrogate data) between the phase (x-axis) of alpha activity and the power (y-axis) of the low gamma activity (30-40Hz) and higher gamma activity (65–90 Hz). The frequency range of the higher gamma activity was consistent with that induced by the stimuli [33]. (C), Normalized time-frequency representations of power for epochs of 160ms time-locked to the peaks of the 12Hz activity for memory item (left) and distractor (right) periods. This approach shows the modulation of low and the induced gamma activity across the alpha cycle during both memory item and distractor processing. However, the alpha phase in which a stronger induced gamma activity is observed differs between the memory item (gamma stronger during the peak) and the distractor processing periods (gamma stronger during the trough). (D) Mean gamma (65-90Hz) power (not normalized) during a 20ms window around the peak and the trough of alpha activity during the memory item and distractor periods (the error bars represent the standard error of the mean across subjects). This graphs shows that gamma activity is stronger during the peak than the trough for the memory item period while the opposite is observed for the distractor period. Interestingly, similar mechanisms could be operating in the hippocampus. A recent study has shown that the theta phase at which gamma activity occurred in the left hippocampus reflected whether or not a given word to be encoded contextually matched or not a picture shown in the background [50].
(TIF)

S1 File (DOCX)
