# Supplementary figures and images for: Gamma Activity Coupled to Alpha Phase as a Mechanism for Top-Down Controlled Gating
Source: PLoS One. 2015 Jun 3;10(6):e0128667. doi: 10.1371/journal.pone.0128667 (PMC4454652; doi:10.1371/journal.pone.0128667)

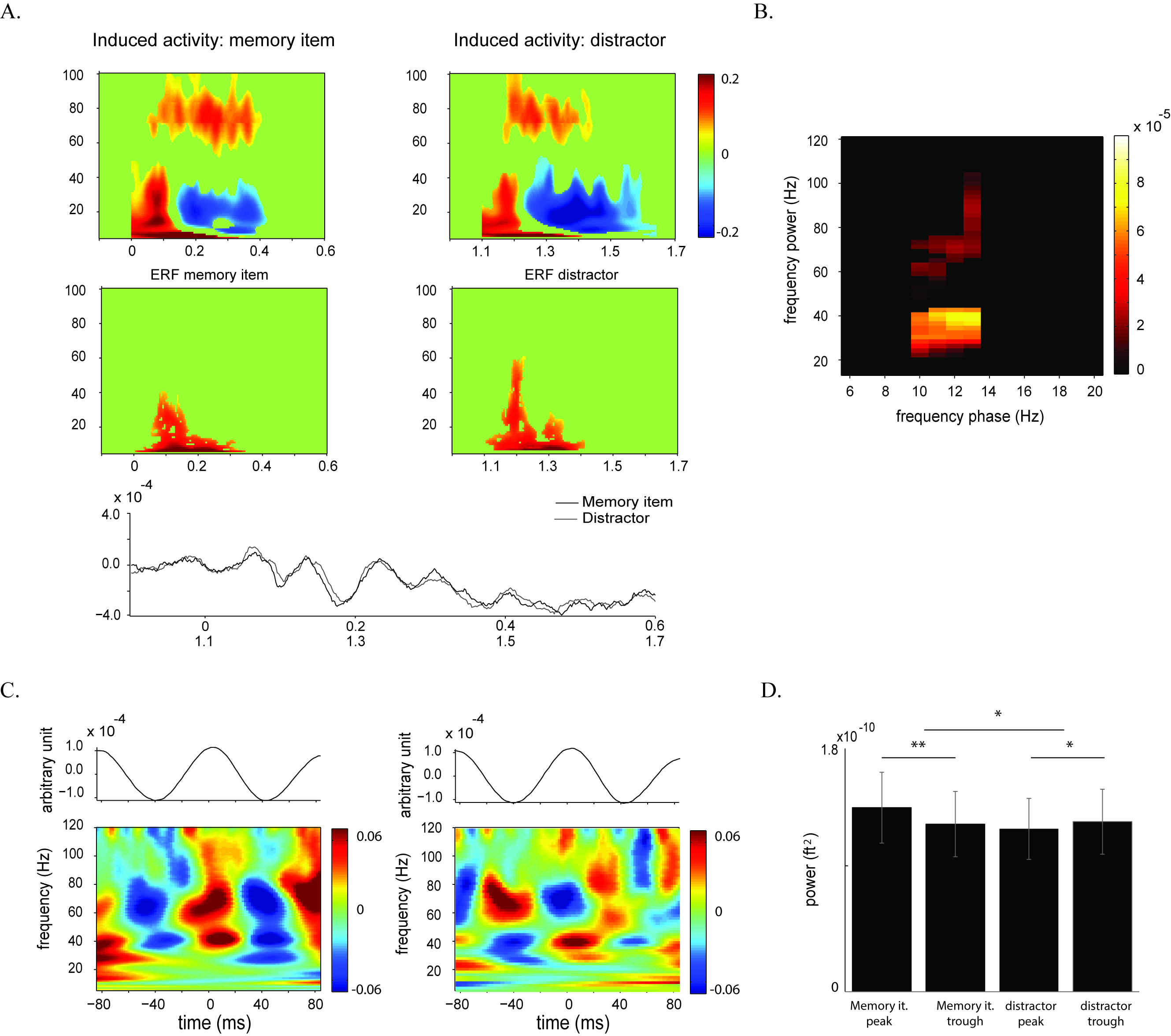

Supplement: S1 Fig — (A) (top) Time-frequency analysis. This analysis indicated that the presentation of the memory item or of the distractor both induces an increase of gamma power in the 65-90Hz band and a decrease of alpha/beta power (8-30Hz) compared to a baseline period defined 200ms before the probe (middle) Time-frequency analysis of the evoked activity. This analysis revealed a strong evoked activity in a large frequency band. Only the activity in the theta and alpha band was sustained up to 300ms after the stimulus onset (bottom) Non-filtered evoked activity. The activity evoked by the memory item and distractor appeared similar. (B) Modulation index (MI) analysis (stimuli periods; memory item: 0.1–0.45s, distractor: 1.2–1.55s). The analysis of the MI indicated a significant coupling (compared to the surrogate data) between the phase (x-axis) of alpha activity and the power (y-axis) of the low gamma activity (30-40Hz) and higher gamma activity (65–90 Hz). The frequency range of the higher gamma activity was consistent with that induced by the stimuli [33]. (C), Normalized time-frequency representations of power for epochs of 160ms time-locked to the peaks of the 12Hz activity for memory item (left) and distractor (right) periods. This approach shows the modulation of low and the induced gamma activity across the alpha cycle during both memory item and distractor processing. However, the alpha phase in which a stronger induced gamma activity is observed differs between the memory item (gamma stronger during the peak) and the distractor processing periods (gamma stronger during the trough). (D) Mean gamma (65-90Hz) power (not normalized) during a 20ms window around the peak and the trough of alpha activity during the memory item and distractor periods (the error bars represent the standard error of the mean across subjects). This graphs shows that gamma activity is stronger during the peak than the trough for the memory item period while the opposite is observed for the distracto [file pone.0128667.s001.tif]
