# Supplementary material for: Gamma Activity Coupled to Alpha Phase as a Mechanism for Top-Down Controlled Gating
Source: PLoS One. 2015 Jun 3;10(6):e0128667. doi: 10.1371/journal.pone.0128667 (PMC4454652; doi:10.1371/journal.pone.0128667)
Supplement: S1 File — (DOCX) [file pone.0128667.s002.docx]

**Supplement: Stimulus processing periods**

**Method**

**Power analyses**

We performed a time-frequency analysis of the activity induced by the memory item and the distractor (-0.1-0.5s and 1-1.6s; common baseline: 2-2.2s) to determine whether we could observe induced gamma activity in both conditions [[1](#_ENREF_1)]. We used a fast Fourier transform (FFT) approach and a sliding time window (5 cycles long; e.g. ΔT=5/f; ΔT = 500 ms for 10 Hz). A Hanning taper (ΔT long) was multiplied to the data prior to the Fourier transform.

The statistical analyses associated with the power analyses were performed using a cluster-based permutation test [[2](#_ENREF_2)] which controls for multiple comparison issue. Significant clusters (i.e. set of significant adjacent points) in the frequency-frequency space were detected using a parametric test-statistic thresholded by an uncorrected p-value of 0.05. Then values in frequency-frequency tiles were reshuffled (500 times) randomly between the actual and the surrogate data and the maximum cluster size per permutation was stored to assess the distribution of maximal cluster sizes. Cluster size was defined as the sum of the t-values in that cluster. Cluster significance in a given contrast was assessed by comparing the cluster size with the distribution of the maximal cluster sizes across permutations. A cluster was considered to be significant at alpha=0.05. This test controls for multiple comparisons since each cluster-level statistic is compared to the reference permutation distribution of the largest cluster-level statistic.

**Cross frequency coupling analysis**

We ran Modulation index and alpha peak-locked time frequency analyses (see method of the main text) for the time window 0.1 to 0.45s for the last memory item and 1.2 to 1.55s for the distractor.

As during the pre-distractor period, we observed that the alpha phase at which the low gamma activity (30-40Hz) was higher differed over subjects. During this period, 8 subjects exhibited a stronger gamma activity during the trough while only 4 subjects exhibited such a pattern during the pre-distractor period (2 overlapping subjects for the two periods). We multiplied the raw data by -1 for these subjects (see the method in the main text).

**Results**

**Memory and distractor items induced gamma power are coupled to different alpha phases**

In our task, memory items (only the last memory item was considered) had to be encoded whereas distractor items had to be ignored. We investigated whether this respective encoding versus ignoring was reflected by the phase relationship between alpha oscillations and gamma power.

**Induced and evoked activity**

We first analyzed the stimuli- induced and evoked activity to determine whether we could observe a sustained gamma activity and whether the evoked activity had a similar shape between the two kind of items so that it would not influence any potential coupling differences observed. As shown by S1A Fig. [ [1](#_ENREF_1)], both the memory item and the distractor induced sustained increase in the gamma band (65-90Hz) and decrease in the alpha-beta band (8-40Hz) compared to baseline (common baseline, 100ms before probe onset).

We observed evoked activity between 5 and 40Hz for the memory item and up to 60Hz for the distractor (see S1A Fig. middle). However a direct comparison of the two conditions did not show significant results (but see Bonnefond and Jensen [1] where we observed a slight shift in the peak of frequency between the two conditions in another brain region with one additional subject). The evoked activity in the alpha-band appeared sustained up to 300ms after stimulus onset. The shape of the unfiltered evoked activity in the two conditions is shown S1A Fig. (bottom).

**Cross-Frequency coupling measures and alpha peak aligned TFRs**

We found a significant coupling between alpha phase (9-13Hz) and the gamma power in the 30-40Hz and in the 65-90Hz frequency band for the stimulus periods (average of the MI observed for the memory item and the distractor periods; see S1B Fig.) when combining the 0.35s interval after the last memory items or distractors. The coupling between these two frequency bands and alpha activity remained significant (p < 0.01) if we compared these values to the values obtained after shuffling the labels of the trials for the phase data (see Statistical analyses in the method section) indicating that these coupling were not due to e.g. the shape of the evoked field.

We then calculated the TFRs aligned to the phase of the alpha activity (S1C Fig.). After normalizing per frequency bin, this allowed to compare the phase relationship for memory versus distractor presentation.

The analysis revealed that the preferred alpha phase of the 65-90 Hz gamma activity was different comparing memory and distractor item processing. To quantify this effect, we extracted the power of gamma activity in a window of 20ms during the peak and the trough of alpha activity detected (see S1D Fig.). We then conducted a two (stimulus type: memory item vs. distractor) by two (alpha phase: alpha peak vs. alpha trough) ANOVA. This analysis revealed a main effect of the stimulus type (F(1,16) = 5, p < 0.05), the gamma power was lower during distractor processing than during memory item processing [[1](#_ENREF_1)]. It also showed an interaction between the stimulus type and the alpha phase (F(1,17) = 11.8, p < 0.001). Post hoc tests revealed a stronger gamma power at alpha peak than at alpha trough for the memory item (p < 0.01) while the reverse was observed for the distractor (p < 0.05).

We further compared trials with low alpha power and trials with high alpha power during the memory item processing and during the distractor processing. However, we did not find any significant effect.

In short, we have observed that the coupling between gamma power and alpha phase differed between memory items to be encoded and distractor items to be ignored.

To further determine whether the gamma activity was coupled to the evoked alpha, we averaged the gamma activity between 65-90Hz during stimulus presentation (Memory item: 0.075-0.4s; distractor; 1.175-1.4s) and subtracted this averaged value (baseline) to the gamma power observed at each frequency and time point within the same time window (procedure similar to the one used for the peak-locked TFR). We did not observe a significant change of gamma activity in these time windows compared to baseline.

**Discussion**

We observed a shift of the preferred alpha phase of the gamma power between the memory encoding and the distractor processing interval (see S1 Fig.). This result could be related to the ideas developed in Jensen et al. [[3](#_ENREF_3)] regarding the role of alpha activity for ordering the processing of relevant and non-relevant items (see also the discussion section in the main text).

Alpha/gamma coupling during anticipation and stimulus processing seem to be quite different. Besides the seemingly distinct gamma frequency coupled to alpha phase, two aspects set the coupling apart: 1) During anticipation we observed a phase-dependent negative correlation between alpha and gamma power. Such negative correlation was not observed during stimulus processing. As such we only found proof-of-principle for the pulsed inhibition notion in the anticipatory interval. 2) The alpha phase at which we observe the negative correlation between alpha power and gamma power, i.e. the phase potentially associated with the pulses of inhibition (see Fig. 3B), matched the phase at which gamma power was relatively high during distractor processing (see Fig. 3A left). Moreover, in some subjects where gamma activity was stronger at the alpha trough, this phase relationship was reversed in the pre-stimulus period (see method section in the main text). This discrepancy suggests that several alpha generators are involved.

In future work it would be highly interesting to further investigate the difference and similarities in the alpha-gamma coupling during respectively anticipation and stimulus processing.

**References**

1. Bonnefond M, Jensen O. The role of gamma and alpha oscillations for blocking out distraction. Commun Integr Biol. 2013;6(1):e22702.
2. Maris E, Oostenveld R. Nonparametric statistical testing of EEG- and MEG-data. J Neurosci Methods. 2007;164(1):177-90.
3. Jensen O, Gips B, Bergmann TO, Bonnefond M. Temporal coding organized by coupled alpha and gamma oscillations prioritize visual processing. Trends Neurosci. 2014;37(7):357-69.
